# Evaluation of radiation dose reduction in head CT using the half-dose method

**DOI:** 10.1007/s11604-023-01410-5

**Published:** 2023-03-24

**Authors:** Yoshitomo Nakai, Osamu Miyazaki, Masayuki Kitamura, Rumi Imai, Reiko Okamoto, Yoshiyuki Tsutsumi, Mikiko Miyasaka, Hideki Ogiwara, Hiroshi Miura, Kei Yamada, Shunsuke Nosaka

**Affiliations:** 1 Department of Radiology, National Center for Child Health and Development, 2-10-1 Okura, Setagaya-Ku, Tokyo, Japan; 2 Department of Radiology, Kyoto Prefectural University of Medicine, 465 Kawaramachi Hirokoji Kamigyo-Ku, Kyoto, Japan; 3 Division of Neurosurgery, National Center for Child Health and Development, 2-10-1 Okura, Setagaya-Ku, Tokyo, Japan; 4 Department of Radiology Japanese Red Cross Society, Kyoto Daini Hospital, 355-5 Haruobicho Kamanza-Dori Marutamachi-Agaru, Kamigyo-Ku Kyoto, Japan

**Keywords:** Computed tomography, CT dose index volume, Dose-length product, Hydrocephalus, Craniosynostosis

## Abstract

**Purpose:**

The present study introduced the half-dose method (HDM), which halves the radiation dose for conventional head computed tomography (CT), for postoperative hydrocephalus and follow-up for craniosynostosis at a children’s hospital. This study aimed to evaluate the contribution of selective head CT scanning optimization towards the overall reduction of radiation exposure.

**Materials and methods:**

We retrospectively assessed 1227 and 1352 head CT examinations acquired before and after the introduction of the HDM, respectively, in children aged 0–15 years. The radiation exposure was evaluated using the CT dose index volume (CTDI-vol), dose-length product (DLP), rate of HDM introduction, and effect of reducing in-hospital radiation dose before and after the introduction of the HDM. For an objective evaluation of the image quality, head CT scans acquired with HDM and full-dose method (FDM) were randomly selected, and the image noise standard deviation (SD) was measured for each scan. In addition, some HDM images were randomly selected and independently reviewed by two radiologists.

**Results:**

The HDM was introduced in 27.9% of all head CTs. The mean CTDI-vol of all head CTs was 21.5 ± 6.9 mGy after the introduction, a 14.9% reduction. The mean DLP was 418.4 ± 152.9 mGy.cm after the introduction, a 17.2% reduction. Compared to the FDM images, the noise SD of the HDM ones worsened by almost 0.9; however, none of the images were difficult or impossible to evaluate.

**Conclusion:**

The HDM yielded diagnostically acceptable images. In addition, a change in protocol for only two diseases successfully reduced the patients’ overall radiation exposure by approximately 15%. Introducing and optimizing the HDM for frequently performed target diseases will be useful in reducing the exposure dose for the hospital’s patient population.

## Introduction

In modern medicine, computed tomography (CT) plays a major role in diagnostic imaging and determining the effectiveness of treatment and is an indispensable modality. Japan has the largest number of CTs among Organization for Economic Co-operation and Development countries. Japan also has the largest number of CTs per million people, at 111.5 [1]. According to the results of a past survey, the number of CTs in Japan is equal to the number of convenience store chains [2].

This availability has led to an increase in the number of CT scans. Moreover, the head CT doses for children in Japan are higher than those in Europe and the United States. This is attributed to the large tube current and the substitution of adult protocols for children in Japan [3]. It may also be due to the late introduction of diagnostic reference levels (DRLs) in Japan, in 2015.

Children exhibit more active cell division and have a smaller body size than adults; thus, the exposure dose per organ in children is higher under the same imaging conditions as that in adults. In addition, the average life expectancy after exposure is longer, and the risk of cancer during that period must also be considered.

A Monte Carlo simulation study showed that the effective dose for head CT in neonates is approximately four times higher than that in adults, indicating that neonates are more susceptible to radiation exposure than adults [4]. Pearce et al. reported an approximately threefold increase in the risk of leukemia and brain tumors in young people aged less than 15 years receiving low doses of 50–60 mGy for head CT radiation [5]. Mathews et al. also reported a 24% higher incidence rate of pediatric cancer with CT scans [6].

The frequency of head CT as a percentage of total pediatric CT is high, reported to be as high as 70% in a previous Japanese national survey [7]. To reduce pediatric exposure, protocols optimized for pediatric body size and evaluation purposes are important. Over the past two decades, optimization has been promoted and exposure reduction has been dramatically implemented in chest and abdominal CT as a result of the US Image Gently’s exposure reduction awareness campaign and popularization of the concept of DRLs [8]. In France, owing to the introduction of DRLs, the 75th percentiles of thoracic and abdominal CT were lower by 21%, as compared with those in 2012 and 2015. However, head CT generally requires a sufficient dose to evaluate fine contrast in the brain parenchyma, such as acute stroke and acute cerebral infarction, and its reduction rate is only 12%, as compared with that of trunk CT [9]. In contrast, cases that do not require evaluation of low-contrast lesions in the brain parenchyma can be evaluated at low doses. Typical cases include evaluation of changes in ventricular size over time (e.g., postoperatively for hydrocephalus) and skull morphology (e.g., postoperatively for craniosynostosis). These diseases are also repeated for postoperative follow-up, and there is a risk of tumor growth due to cumulative radiation exposure from frequent CT scans [10].

Selective optimization of protocols for these diseases may lead to a reduction in overall exposure; nonetheless, it remains unclear to what extent the introduction of selective head CT optimization could contribute to the overall reduction of head CT exposure.

The present study introduced the half-dose method (HDM), which uses half the dose for conventional head CT, to evaluate ventricular size after hydrocephalus surgery and the degree of bony fusion in craniosynostosis at a children’s hospital. This study aimed to clarify to what extent the overall exposure dose for simple head CT can be reduced by optimizing the method only for specific diseases.

## Materials and methods

### Study population

This study was approved by the ethics committee of our hospital. HDM has been implemented in our hospital since November 2015. This retrospective study was approved by the institutional review board and the need to obtain written informed patient consent was waived due to the nature of the study.

Participants aged 0–15 years were retrospectively validated on 1227 simple CT head scans taken during the 8 months before the introduction of the HDM (January to August 2015) and 1352 scans taken during the 8 months after the introduction of the HDM (January–August 2016).

### Indication criteria for the half-dose method

The indications for the HDM were as follows: (1) postoperative follow-up examinations for hydrocephalus requested by neurosurgery or emergency departments to confirm ventricular size and drainage catheterization and (2) postoperative follow-up examinations for craniosynostosis to evaluate bone fusion. These two indication criteria were thoroughly discussed beforehand between the radiologists and neurosurgeons and were applied with the neurosurgeons’ approval.

Before each CT scan, the radiologist in charge always reviewed the request in the electronic medical record and decided on the imaging method. Even after hydrocephalus or craniosynostosis, the HDM was not performed in cases before ventricular shunt insertion or osteotomy, and the usual full-dose method (FDM) was used. In addition, when evaluation of other diseases, such as tumor or cerebral hemorrhage, was required, imaging was performed using the usual protocol, FDM, and in cases of doubt, the imaging protocol was determined by the radiologist after consultation with the neurosurgeon.

All requests for head CT other than postoperative follow-up for the two abovementioned diseases and for the comparison group (1227 cases taken from January to August 2015 before the introduction of the HDM) were taken with the FDM.

### Image acquisition and reconstruction parameters

The CT system was a 64-row MDCT (Discovery 750HD; GE Healthcare, Milwaukee, WI, USA). All CT imaging conditions were extracted using the dose optimization solution (DoseWatch; GE Healthcare, Milwaukee, WI, USA).

In addition, image noise was reduced by image reconstruction using Advanced Statistical Iterative Reconstruction (ASiR). The ASiR level setting was reconfigured at 80%.

The tube current was automatically controlled using CT-automatic exposure control, and the maximum tube current was set for FDM and HDM imaging conditions according to the patients’ age group (28 days, 28 days–3 years, 3–6 years, > 6 years.

The HDM reduces exposure by 50% of the maximum tube current for conventional head CT. The maximum tube currents of the FDM for 28 days, 28 days–3 years, 3–6 years, and > 6 years were 130, 210, 220, and 235 mA, respectively, whereas the maximum tube currents of the HDM were 65, 105, 110, and 115 mA, respectively.

The scanning parameters for imaging conditions other than the tube current were as follows: tube voltage, 120 kVp; gantry rotation, 0.4 s; helical pitch, 0.53; and beam width, 20 mm (or 40 mm). The reconstructed slice thickness was set at 5 mm and 2.5 mm for infants. Bone fusion was evaluated by three-dimensional reconstruction created by Centricity Advantage Workstation (GE Healthcare, Milwaukee, WI, USA).

### Image quality analysis

#### Objective evaluation

For image quality analysis, 20 head CT scans taken with HDM and FDM in each age group (< 1 year, 1 to < 5 years, 5 to < 10 years, and 10 to < 15 years) were randomly selected. A radiologist with 9 years of experience established 20-mm^2^ oval-shaped regions-of-interest (ROI) in the ventricles, cerebrum, brainstem, and cerebellum, with uniform image quality. Image noise standard deviation (SD) was measured and compared between FDH and HDM groups using the Wilcoxon rank-sum test.

#### Subjective evaluation

For retrospective analysis, seven patients in each age group imaged with HDM were randomly selected, and images were independently reviewed by two radiologists specializing in children (reader 1: 9 years of radiology experience; reader 2: 33 years of radiology experience). The evaluation structures were compared with the preoperative FDM images of the same patient. The clarity of each of the evaluated structures (ventricles, corticomedullary boundary, extramedullary space, bone suture and fusion in 2D and 3D images) was assessed on a 5-point scale as follows: 5—very well evaluable (as good as FDM), 4—well evaluable (slightly inferior to FDM), 3—evaluable (inferior to FDM), 2—difficult to evaluate, 1—not possible to evaluate.

For a prospective analysis, a diagnostic radiologist assessed the image quality in real time on the console immediately after imaging and evaluated whether the image was diagnostic or not.

#### Dose evaluation

CT doses were evaluated using the CT dose index volume (CTDI-vol) and dose-length product (DLP). These doses were converted and displayed as the equivalent of a 16-cm-diameter CTDI phantom extracted from the dose optimization solution (DoseWatch). From the obtained data, using the boxplot method, the minimum, first quartile (25th percentile), median, third quartile (75th percentile), and maximum values were obtained for the CTDI-vol, and the DLP for both pre- and post-HDM was evaluated.

Patient backgrounds (age, sex, and classification of disease categories) were obtained from clinical information recorded in the reading reports as part of the demographic evaluation for the two groups of patients before and after the introduction of the HDM.

## Data analysis

The age of the participants was classified into four age groups (1 year and younger, 1 to < 5 years, 5 to < 10 years, and 10 to < 15 years), and the distribution was examined. The number of CT scans taken during the study period was extracted for each. HDM implementation rates were calculated by two disease categories and by age group after the HDM was introduced, and trends in CT protocols were analyzed.

Next, we examined the extent to which the introduction of the HDM contributed to the reduction of overall head CT dose. First, to confirm the dose for examinations taken with the HDM, we compared the CTDI-vol and DLP between examinations taken with the HDM and those taken with the FDM during the HDM period. We confirmed that the dose in the HDM group was actually half to that of the FDM group. Subsequently, the CTDI-vol and DLP of all CT scans were compared using the Wilcoxon rank-sum test before and after the introduction of the HDM for all ages and by age group. We also calculated the reduction in radiation dose before and after the introduction of the HDM.

Statistical analyses were performed using JMP® version 14.2.0 (SAS Institute Inc., Cary, NC, USA), with the significance level set at *P* < 0.05.

## Results

### Dose evaluation

Demographics before and after the introduction of the HDM are shown in Table 1. There were 1227 and 1352 whole head CTs in the pre- and post-HDM groups, respectively. There was no sex difference, and the highest percentage of patients in both the pre- and post-HDM groups was between 1 and 5 years of age. Of the HDM cases, 416 and 444 were for hydrocephalus in the pre- and post-HDM groups, respectively, and 78 and 115 were for craniosynostosis in the pre- and post-HDM groups, respectively. The percentages of target diseases were 40% and 41% in the pre- and post-HDM groups, respectively. The average number of CT scans by disease did not change, with greater repeated CT scans after hydrocephalus surgery.

**Table 1:** Demographics and clinical characteristics

	Pre-HDM	Post-HDM
Examination number	1227	1352
Median (range 0–14.9 years)	2.5	2.8
Age class, *n* (%)		
< 1 year	341 (27.8%)	438 (32.4%)
1 to < 5 years	469 (38.2%)	489 (36.2%)
5 to < 10 years	264 (21.5%)	258 (19.1%)
10 to < 15 years	153 (12.5%)	167 (12.4%)
Sex, *n*		
Male, *n*	711	767
Female, *n*	516	585
Disease category, *n*		
Hydrocephalus	416 (33.9%)	444 (32.8)%
Craniosynostosis	78 (6.4%)	115 (8.5%)
Others	733 (59.7%)	793 (58.7%)
Mean number of CT scans (± SD)		
Hydrocephalus	2.3 ± 2.4	2.2 ± 2.4
Craniosynostosis	1.6 ± 1.4	1.7 ± 1.5
Others	1.4 ± 1.2	1.4 ± 1.2

*HDM*, half-dose method, *CT* computed tomography, *SD* standard deviation

**Fig. 1:**
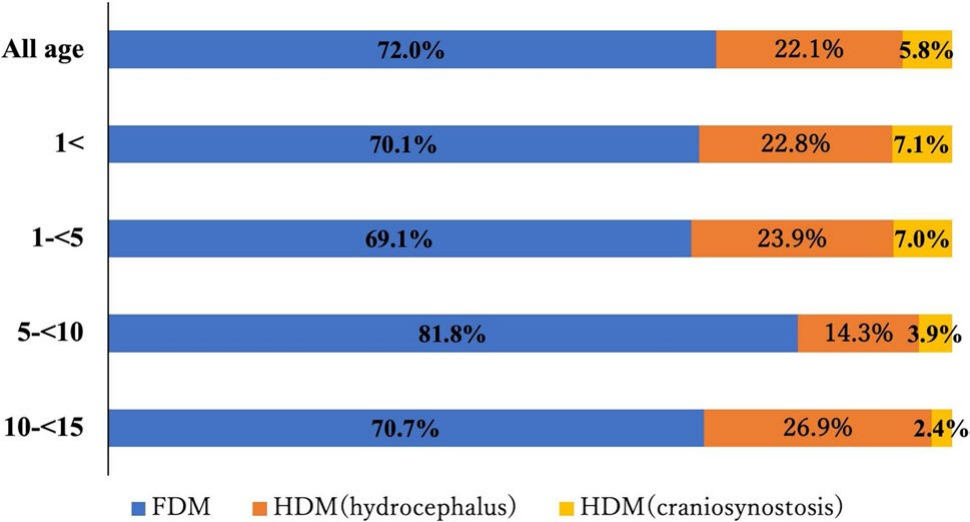
Percentage of CT protocols for each age group. HDM was used in 27.9% of all head CTs. By age, 29.9%, 30.9%, 18.2%, and 29.3% of the patients were aged < 1 year, 1–5 years, 5–10 years, and 10–15 years, respectively

Table 2 presents each CT protocol and disease category and the number and percentage of cases after the introduction of the HDM (1352 cases), whereas Fig. 1 shows the distribution of the percentages graphically. The HDM was used in 28.0% of all head CT, with the most common age group being aged 1– < 5 years (approximately 31%), followed by aged 1 year (approximately 30%).

**Table 2:** Summary of patient distribution in the post-HDM group (*n* = 1352)

Scan protocol	Disease category	Age group, *n* (%)				
		< 1 year	1 to < 5 years	5 to < 10 years	10 to < 15 years	Total
HDM	Hydrocephalus	100 (22.8)	117 (23.9)	37 (14.3)	45 (26.9)	299 (22.1)
	Craniosynostosis	31 (7.1)	34 (7.0)	10 (3.9)	4 (2.4)	79 (5.8)
	Subtotal	131 (29.9)	151 (30.9)	47 (18.2)	49 (29.3)	378 (28.0)
FDM		307 (70.1)	338 (69.1)	211 (81.8)	118 (70.7)	974 (72.0)
Total		438	489	258	167	1352

*HDM* half-dose method, *FDM* full-dose method

**Fig. 2:**
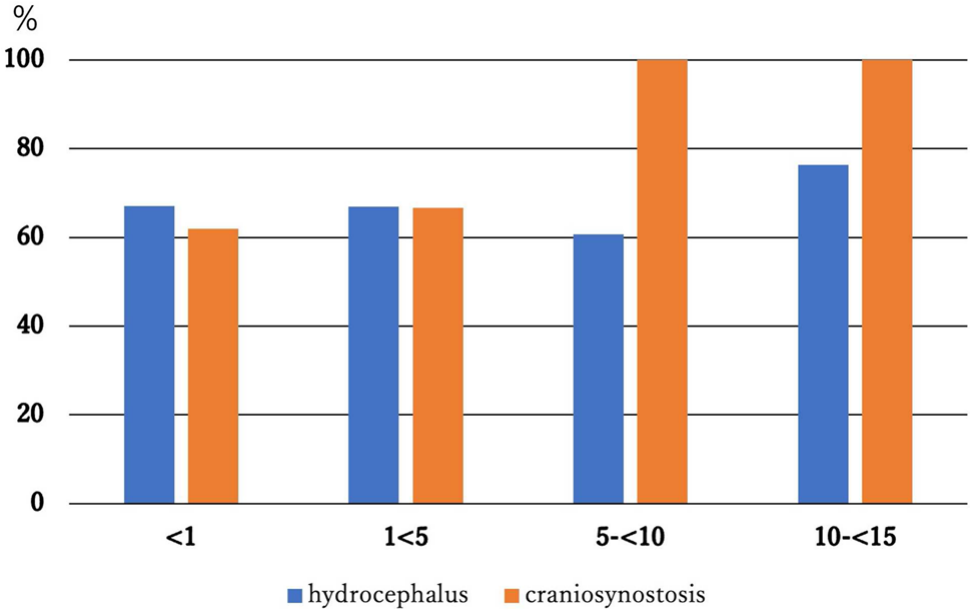
Frequency of the HDM for hydrocephalus and craniosynostosis in each age group (chronological change). The HDM rate for hydrocephalus was approximately 60–70% in all age groups; the HDM rate for craniosynostosis was higher in patients older than 5 years

Table 3 and Fig. 2 show the frequency of the HDM by age group. In hydrocephalus, the induction rate for each age group was approximately 60–70%.

**Table 3:** Implementation rate by disease in each age group

	FDM, *n*	HDM, *n*	HDM implementation rate
Hydrocephalus (All age)	145	299	67.3%
< 1 year	49	100	67.1%
1 to < 5 years	58	117	66.9%
5 to < 10 years	24	37	60.7%
10 to < 15 years	14	45	76.3%
Craniosynostosis (All age)	36	79	68.7%
< 1 year	19	31	62.0%
1 to < 5 years	17	34	66.7%
5 to < 10 years	0	10	100%
10 to < 15 years	0	4	100%

*FDM* full-dose method, *HDM* half-dose method

In contrast, the HDM rate for craniosynostosis increased with age.

**Fig. 3:**
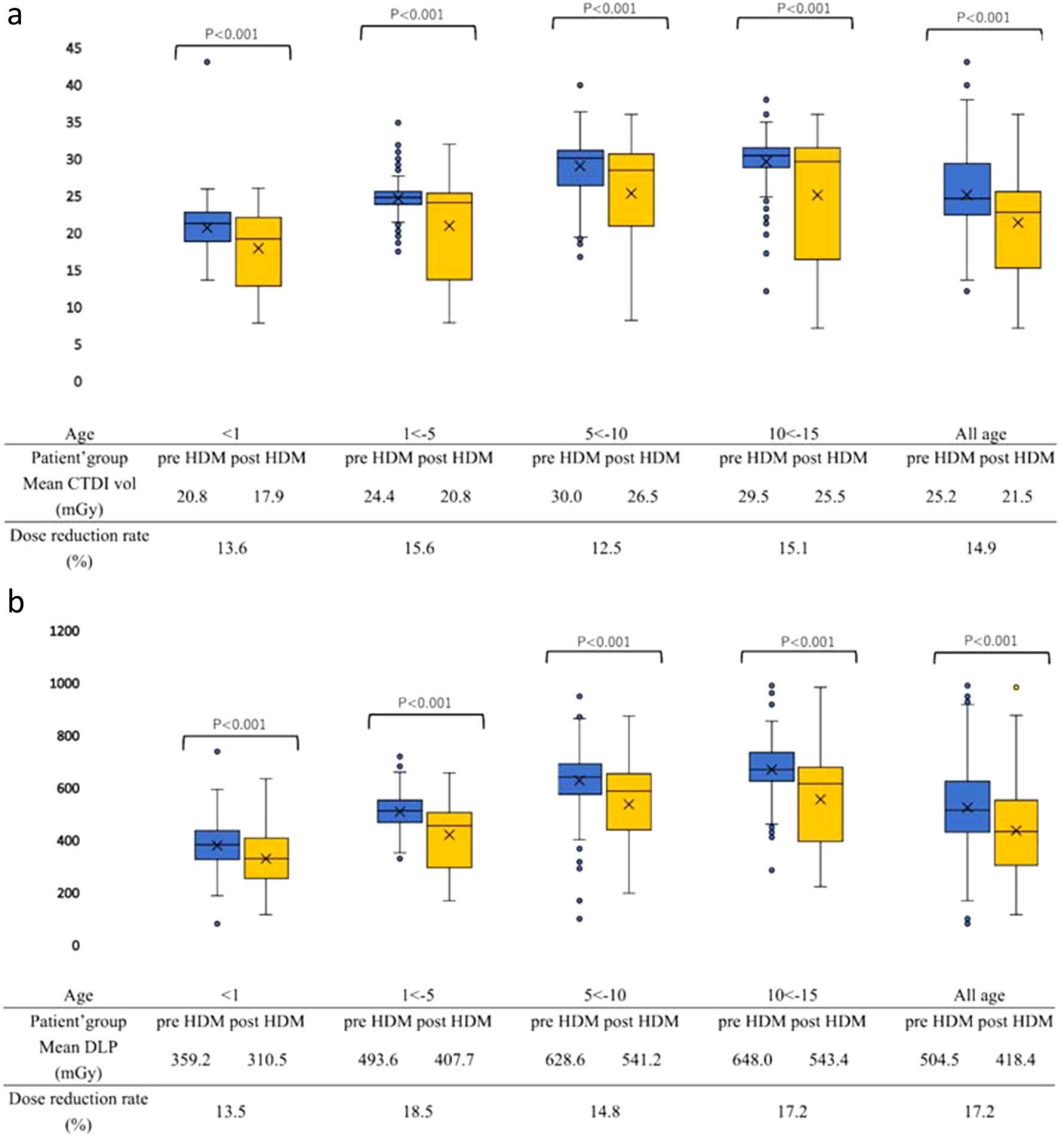
Boxplots for CTDI-vol (**a**) and DLP (**b**) before and after the introduction of the HDM among age groups, as well as the dose reduction rate and statistical evaluation. **a** CTDI-vol (mGy). **b** DLP (mGy.cm). The mean CTDI-vol of head CT for all ages was 25.2 ± 4.5 mGy and 21.5 ± 6.9 mGy in the pre- and post-HDM groups, respectively, a 14.9% decrease before and after the introduction of the HDM. The mean DLPs of all head CTs were 504.5 ± 137.6 mGy.cm and 418.4 ± 152.9 mGy.cm in the pre- and post-HDM groups, respectively, with an overall reduction of 17.2% before and after the introduction of the HDM

Table 4 shows the CTDI-vol and DLP before and after the introduction of the HDM for minimum, 25th percentile, median, 75th percentile, and maximum for all ages and four age groups. The results are also shown in a boxplot in Fig. 3. The mean CTDI-vol of all head CTs were 25.2 ± 4.5 mGy and 21.5 ± 6.9 mGy before and after the introduction of HDM, respectively, a decrease of 14.9%. The mean DLP of all head CTs were 504.5 ± 137.6 mGy.cm and 418.4 ± 152.9 mGy.cm in the pre-HDM and post-HDM groups, respectively; a decrease of 17.2% before and after HDM introduction. A similar reduction in radiation exposure was observed in all age groups (Fig. 3). For all ages, CTDI-vol and DLP were statistically significantly lower in the post-HDM group than in the pre-HDM group (*P* < 0.001).

**Table 4:** Comparison of CTDI-vol and DLP before and after the introduction of the HDM

		CTDI-vol		DLP	
		Pre-HDM	Post-HDM	Pre-HDM	Post-HDM
Total	Maximum	43.0	36.0	967.3	962.8
	75th percentile	29.3	25.6	604.2	531.6
	Median	24.6	22.7	495.4	412.0
	25th percentile	22.5	15.3	408.4	282.2
	Minimum	12.2	7.1	59.3	95.2
	Mean ± SD	25.2 ± 4.5	21.5 ± 6.9	504.5 ± 137.6	418.4 ± 152.9
< 1 year	Maximum	43.0	26.0	715.6	612.3
	75th percentile	22.8	20.1	415.5	386.0
	Median	21.3	19.2	360.9	307.9
	25th percentile	18.9	12.3	305.7	231.2
	Minimum	13.6	7.8	59.3	95.2
	Mean ± SD	20.8 ± 3.2	17.9 ± 4.9	359.2 ± 83.2	310.5 ± 94.4
1 to < 5 years	Maximum	34.5	31.6	716.6	636.8
	75th percentile	25.3	25.0	530.9	484.7
	Median	24.4	23.7	490.7	433.4
	25th percentile	23.5	13.3	447.0	273.4
	Minimum	17.1	7.5	307.0	148.9
	Mean ± SD	24.4 ± 1.9	20.8 ± 5.8	493.6 ± 66.9	407.7 ± 117.5
5 to < 10 years	Maximum	39.9	36.0	929.2	851.1
	75th percentile	31.2	30.7	671.5	631.8
	Median	30.1	28.5	621.2	567.0
	25th percentile	26.4	21.0	552.2	418.7
	Minimum	16.8	8.2	79.8	177.3
	Mean ± SD	30.0 ± 2.8	26.5 ± 6.6	628.6 ± 97.5	541.2 ± 138.1
10 to < 15 years	Maximum	38.0	36.0	967.3	962.3
	75th percentile	31.5	31.5	714.2	656.7
	Median	30.5	29.7	647.1	594.2
	25th percentile	28.8	16.4	603.0	375.9
	Minimum	12.2	7.13	263.0	200.1
	Mean ± SD	29.5 ± 3.6	25.5 ± 8.1	648.0 ± 108.2	543.4 ± 161.6

*CTDI-vol* computed tomography dose index volume, *DLP* dose-length product, *HDM* half-dose method, *SD* standard deviation

### Image quality analysis

All images taken were evaluated by a pediatric radiologists and neurosurgeons, and none of the cases required another CT scan due to poor image quality.

#### Objective evaluation

Compared to that in the FDM images, the noise SD was worsened by 0.8 in the ventricles, 1.0 in the cerebrum, 0.9 in the brainstem, and 0.9 in the cerebellum in HDM images. Overall, the difference in SD worsened by 0.9 (Table 5).

**Table 5:** Comparison of image noise (SD) between FDM and HDM

		Ventricle			Cerebrum		
		FDM	HDM	*P* value	FDM	HDM	*P* value
Mean ± SD	1 < year	2.2 ± 0.6	3.0 ± 0.7	< 0.05	3.0 ± 0.6	4.1 ± 1.0	< 0.05
	1 to < 5 years	2.7 ± 0.6	3.4 ± 0.6	< 0.05	3.5 ± 0.7	4.5 ± 0.9	< 0.05
	5 to < 10 years	2.5 ± 0.4	3.7 ± 0.8	< 0.05	3.5 ± 0.6	4.2 ± 0.7	< 0.05
	10 to < 15 years	2.9 ± 0.6	3.5 ± 0.8	< 0.05	3.2 ± 0.7	4.5 ± 0.6	< 0.05
	All age	2.6 ± 0.6	3.4 ± 0.7	< 0.05	3.3 ± 0.6	4.3 ± 0.8	< 0.05
		Brain stem			Cerebellum		
		FDM	HDM	*P* value	FDM	HDM	*P* value
	< 1 year	3.2 ± 0.8	3.8 ± 0.8	< 0.05	3.0 ± 0.8	3.6 ± 0.8	< 0.05
	1 to < 5 years	3.6 ± 0.6	4.5 ± 0.7	< 0.05	3.1 ± 0.5	4.3 ± 0.7	< 0.05
	5 to < 10 years	3.3 ± 0.6	4.6 ± 0.6	< 0.05	3.4 ± 0.7	4.0 ± 0.6	< 0.05
	10 to < 15 years	3.7 ± 0.8	4.7 ± 0.9	< 0.05	3.2 ± 0.5	4.4 ± 0.7	< 0.05
	All age	3.5 ± 0.7	4.4 ± 0.8	< 0.05	3.2 ± 0.7	4.1 ± 0.8	< 0.05

*FDM* full-dose method, *HDM* half-dose method, *SD* standard deviation

#### Subjective evaluation

**Fig. 4:**
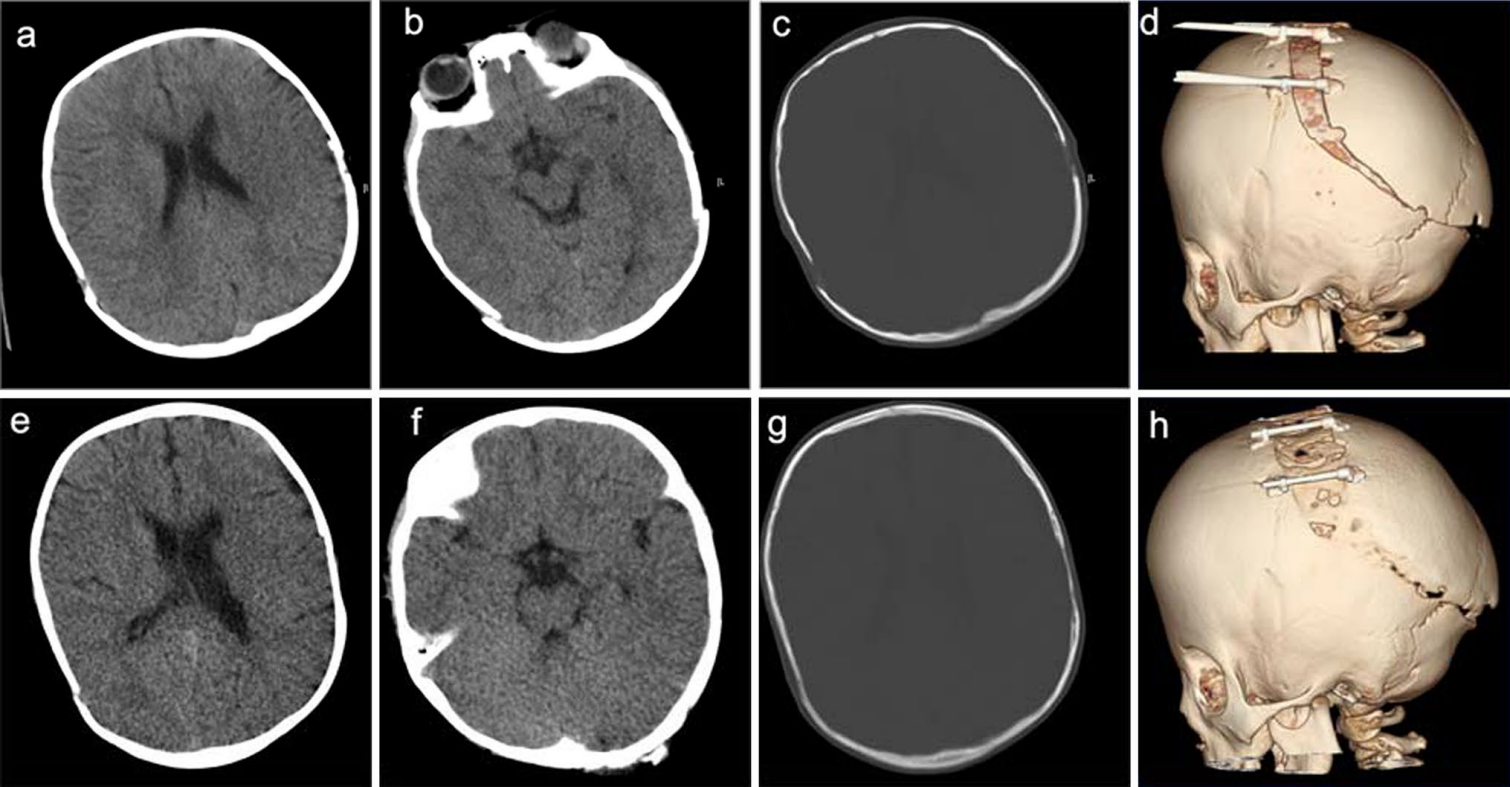
Example of comparison: four categories of view-points in the half-dose method (HDM) and full-dose method (FDM). A 4-year-old female with coronal suture craniosynostosis. Cranial computed tomography (CT) image obtained soon after cranioplasty by the FDM (**a**–**d**), and follow-up CT imaged obtained using the HDM at 4 months after the previous CT (**e**–**h**). FDM scan parameters: tube voltage, 120 kV; tube current, 220 mA; CTDI-vol, 24.4 mGy; DLP, 589.1 mGy・cm. HDM scan parameters: tube voltage, 120 kV; tube current, 130 mA; CTDI-vol, 12.5 mGy; DLP, 257.8 mGy・cm. In terms of image quality, the brain parenchyma on images obtained by the HDM (**e**) was relatively coarse as compared to that on images obtained by the FDM (**a**). However, the corticomedullary boundary of the cerebral hemisphere can be recognized on the HDM image (**e**). Additionally, the ventricular system (**a**, **e**), and 2D (**c**, **g**) and 3D (**d**, **h**) cranium images seemed to have a similar image quality in HDM and FDM images, without loss of diagnostic value

Ventricles and bones were well evaluable in all cases. Although there were differences in the corticomedullary boundary and extramedullary space trends among examiners, all regions could be evaluated, and no cases were difficult or impossible to evaluate (Fig. 4; Table 6).

**Table 6:** Subjective evaluation of the half-dose method

		Ventricle		Corticomedullary boundary		Extramedullary space	
	Scale	Reader 1	Reader 2	Reader 1	Reader 2	Reader 1	Reader 2
N (%)	5	24 (85.7%)	24 (85.7%)	1 (3.6%)	20 (71.4%)	14 (50%)	23 (82.1%)
	4	4 (4.3%)	4 (4.3%)	24 (85.7)	8 (28.6%)	14 (50%)	5 (17.9%)
	3	0 (0%)	0 (0%)	3 (10.7)	0 (0%)	0 (0%)	0 (0%)
	2	0 (0%)	0 (0%)	0 (0%)	0 (0%)	0 (0%)	0 (0%)
	1	0 (0%)	0 (0%)	0 (0%)	0 (0%)	0 (0%)	0 (0%)
		Cranium 2D		Cranium 3D			
	Scale	Reader1	Reader2	Reader1	Reder2		
	5	28 (100%)	27 (96.4%)	28 (100%)	27 (96.4%)		
	4	0 (0%)	1 (3.6%)	0 (0%)	1 (3.6%)		
	3	0 (0%)	0 (0%)	0 (0%)	0 (0%)		
	2	0 (0%)	0 (0%)	0 (0%)	0 (0%)		
	1	0 (0%)	0 (0%)	0 (0%)	0 (0%)		

5—very well evaluable (as good as FDM), 4—well evaluable (slightly inferior to FDM), 3—evaluable (inferior to FDM), 2—difficult to evaluate, 1—not possible to evaluate

Reader 1: 9 years of radiology experience; Reader 2: 33 years of radiology experience)

## Discussion

We succeeded in reducing overall head CT radiation exposure to approximately 15% by introducing HDM for postoperative hydrocephalus and craniosynostosis.

Hydrocephalus and craniosynostosis tend to cause repeated CT scans in children; as a contributing factor, patients after hydrocephalus shunting surgery have a high frequency of complications, such as shunt dysfunction, overdrainage, and infection, with 30–40% failing during the first year after surgery [11].

This may also be because follow-up is essential to evaluate bone fusion even after surgery for craniosynostosis [12]. The results of this study also showed that the majority of the patients with hydrocephalus had a shunt placed and were under long-term follow-up. The frequency of repeat CT scans for hydrocephalus was higher than for craniosynostosis.

Although HDM achieves a low radiation dose by halving the current, the CT images taken at low doses have increased image noise. However, all subjective evaluations in this study indicated good image quality with respect to ventricular size and bone evaluation, and no problems were observed in the evaluation of the corticomedullary boundary and intraparenchymal space. In addition, the results of the present study demonstrated that there were no cases among the 378 patients in which poor HDM image quality necessitated repeated examination, proving that clinical application of the method is not problematic.

In the evaluation of ventricular size, shunt tube tip position, and bony fusion required in the postoperative follow-up of hydrocephalus and craniosynostosis, the delineation of fine structures, such as the contrast of the corticomedullary junction and basal ganglia, is not important. In the past, several reports have been published on radiation dose reduction for hydrocephalus and craniosynostosis, and Gabriel et al. reported that the size of the ventricles could be evaluated after hydrocephalus surgery even at a dose 90% lower than that routinely used [13]. They also reported that in craniosynostosis, 83% and 88% reduced doses in neonatal and 5-year-old phantoms could be used to evaluate the size of the ventricles without compromising image quality [14]. These methods were reported using simulations and phantoms, and there are few reports on the degree of contribution to the overall reduction of head CT radiation exposure in actual clinical practice using a targeted disease-specific radiation reduction protocol. However, as mentioned above, a 50% reduction in the HDM does not significantly degrade image quality and is considered to be applicable in actual clinical practice.

In the literature, apart from the follow-up of hydrocephalus and craniosynostosis, imaging at approximately 45% of the normal dose in the follow-up of cerebral hemorrhage has been adequate for evaluation [15]. Other low-dose imaging for the fine bone structures of the temporal bone and sinus cavities has also been reported [16, 17]. Although careful judgment is required in evaluating image quality, further reduction of radiation exposure is expected if the application of such optimized low-dose protocols is expanded. Miglioretti et al. estimated that optimizing the imaging conditions of CT examinations above the 75th percentile of the dose distribution corresponding to the DRL to the median in the United States would reduce the incidence of CT-induced cancer by 43% [18]. Journey et al. also estimated that if the dose for individual CT examinations could be optimized and reduced by 20% and 40% in the United Kingdom, CT-induced cancer could also be reduced by 20% and 40% [19]. Based on these literature discussions, the introduction of the HDM for only two diseases in this study can also reduce the risk of CT-induced cancer by approximately 15% at our institution.

If each facility implements low-dose protocols and optimizes imaging for each examination, this will lead to a reduction in radiation dose for the country as a whole in the future, leading to a decrease in DRLs and a reduction in future cancer risk.

The role of the radiologist is to evaluate images and optimize examinations according to the “as low as reasonably achievable” principle, and it is an important responsibility to reduce future risks due to radiation exposure.

## Limitations

This study has some limitations. First, this was a single-facility study. Our facility is a specialized children’s hospital, and the distribution of diseases may differ from that observed in a general hospital. Second, although the indications were limited to hydrocephalus and craniosynostosis, it may be possible to also include the HDM in the preoperative period. In addition, expansion of the indications for the HDM should be considered in the future. Moreover, there remains the challenge of further reducing the dose by 60% or 70%, rather than halving the dose.

## Conclusion

The introduction of the HDM with a 50% reduction in radiation exposure for diseases that do not require high doses but require repeated CT scans, including in patients after hydrocephalus shunting or craniosynostosis surgery, has successfully reduced head CT exposure by approximately 15% for the entire hospital. As each hospital adopts low-dose protocols, such as the HDM, the risk of exposure is expected to be reduced.
